# Supplementary material for: Comparing a Query Compound with Drug Target Classes Using 3D-Chemical Similarity
Source: Int J Mol Sci. 2020 Jun 12;21(12):4208. doi: 10.3390/ijms21124208 (PMC7352980; doi:10.3390/ijms21124208)
Supplement: Supplementary file 1 [file ijms-21-04208-s001.zip › 2.Supporting_Informations.docx]

**Supporting Information**

**Comparing a query compound with drug target classes using 3D- chemical similarity**

Sanghyeok Lee, ^1^ Sangjin Ahn, ^2^ Mi-hyun Kim^1^*

*^1^Gachon Institute of Pharmaceutical Science and Department of Pharmacy, College of Pharmacy, Gachon University, Yeonsu-gu, Incheon, 21936, Republic of Korea, ^2^Department of Financial Engineering, College of Business, Ajou University, Suwon, 16499, Republic of Korea*

^*^Author for correspondence

E-mail: [kmh0515@gachon.ac.kr](mailto:kmh0515@gachon.ac.kr)

**Table of Contents**

1. Mathematical description
2. Summary of datasets (S.Table1)
3. The common ligands between target classes (S.Table2)
4. 2D Structures of queries (S.Table3)
5. Gaussian mixture models for additional 4 target classes (S.Figures 1 to 4)
6. Frequency distributions of $\Xi_{ML}$ estimates ($\mu_{1}$and$\sigma_{1}$) for target classes (S.Figures 5 to 7)
7. Python script for 3D similarity matrix

**The derivation of Kullback–Leibler (K–L) divergence between the two Gaussian distributions.**

The Kullback–Leibler divergence, $D(P|\left| Q \right),$ is defined as either

$D\left( P\left\| Q \right. \right) := \int_{-\infty}^{\infty} \ln\left( \frac{p\left( x \right)}{q\left( x \right)} \right)p\left( x \right)dx$, (S1.1)

We assume the probability distributions P(x) and Q(x) replace the Gaussian distributions $G\left( x;m_{i}, \sigma_{i} \right)$and $G\left( x;m_{j}, \sigma_{j} \right)$, where

$G\left( x;m_{i}, \sigma_{i} \right):= \int_{-\infty}^{x} g(s;m_{i},\sigma_{i})ds$ and $G\left( x;m_{j}, \sigma_{j} \right):= \int_{-\infty}^{x} g(s;m_{j},\sigma_{j})ds$, (S1.2)

and the probability density functions, $g(s;m_{i},\sigma_{i})$ and $g(s;m_{j},\sigma_{j})$, are

$\left\{ \begin{aligned} g\left( s;m_{i},\sigma_{i} \right)= \frac{1}{\sigma_{i}\sqrt{2\pi}}\exp\left( -\frac{\left( s-m_{i} \right)^{2}}{2\left( \sigma_{i} \right)^{2}} \right) \\ g\left( s;m_{j},\sigma_{j} \right)=\frac{1}{\sigma_{j}\sqrt{2\pi}}\exp\left( -\frac{\left( s-m_{j} \right)^{2}}{2\left( \sigma_{j} \right)^{2}} \right) \end{aligned} \right.$ . (S1.3)

Using (S1.1) and (S1.3), the Kullback–Leibler divergence between the two Gaussian distributions $G\left( x;m_{i}, \sigma_{i} \right)$ and $G\left( x;m_{j}, \sigma_{j} \right)$ in (S1.2) are as follows:

$D\left( G\left( x;m_{i}, \sigma_{i} \right)\left\| G\left( x;m_{j}, \sigma_{j} \right) \right. \right)$

=$\text{}_{i}\left[ \ln\left( \frac{G\left( x;m_{i}, \sigma_{i} \right)}{G\left( x;m_{j}\text{,}\sigma_{j} \right)} \right) \right]$

= $\int_{-\infty}^{\infty} g(s;m_{i},\sigma_{i})\ln(g\left( x;m_{i},\sigma_{i} \right))ds- \int_{-\infty}^{\infty} g(s;m_{i},\sigma_{i})\ln(g\left( x;m_{j},\sigma_{j} \right))ds$

$=\int_{-\infty}^{\infty} g(s;m_{i},\sigma_{i})\ln\left( -\frac{\left( s-m_{i} \right)^{2}}{{2\left( \sigma_{i} \right)}^{2}}-\ln\left( \sigma_{i}\sqrt{2\pi} \right) \right)ds+ \int_{-\infty}^{\infty} g(s;m_{i},\sigma_{i})\ln(-\frac{\left( s-m_{j} \right)^{2}}{{2\left( \sigma_{j} \right)}^{2}}+\ln(\sigma_{j}\sqrt{2\pi}))ds$. (S1.4)

By the following relationships,

$\int_{-\infty}^{\infty} g\left( s;m,\sigma\right)ds=1,$

$\int_{-\infty}^{\infty} sg\left( s;m,\sigma\right)ds=m,$ (S1.5)

$\int_{-\infty}^{\infty} \left( s-m \right)^{2}g\left( s;m,\sigma\right)ds=\left( \sigma\right)^{2},$

we obtain

$$=-\frac{1}{2}-\ln(\sigma_{i}\sqrt{2\pi})+ \frac{\left( \sigma_{i} \right)^{2}+\left( m_{i}-m_{j} \right)^{2}}{2\left( \sigma_{j} \right)^{2}\text{ }}+\ln(\sigma_{j}\sqrt{2\pi})$$

$=\ln\left( \frac{\sigma_{j}}{\sigma_{i}} \right)+\frac{{(\sigma_{i})}^{2}+\left( m_{i}-m_{j} \right)^{2}}{2{(\sigma_{j})}^{2}\text{ }}-\frac{1}{2}$ . (S1.5)

**The mathematical elucidation of expectation-maximization (EM) algorithm to achieve the hyperparameters of Gaussian mixture model (GMM).**

The mixture models, $\Xi\left( \Phi\left( \mathbf{x} \right) | p, K \right),$ is defined as

$\Xi\left( \Phi\left( \mathbf{x} \right) | p,\omega, \lambda, K \right):= \sum_{k=1}^{K} \omega_{k} p\left( \mathbf{x} : \lambda_{k} \right)$ (S1.6)

with $\Phi$ representing a probabilistic density generated from the unknown compositional data, $p$ representing a well-known probability density, and **x** representing a random vector, the functional operator.

Concretely, the EM algorithm for GMM is summarized as follows:

- Start with an initial guess for the GMM parameters.

- E-step: Calculate the conditional expectation of the log likelihood, $L_{h}()$, for the incomplete data $\Phi\left( x \right)$ with respect to $\sum_{k=1}^{K} \hat{\omega}_{k}^{\left( n \right)} g\left( \mathbf{x};\hat{m}_{k}^{\left( n \right)},\hat{\sigma}_{k}^{\left( n \right)} \right)$, $Q_{\boldsymbol{\Phi}}$, and

$\int_{-\infty}^{\infty} \sum_{k=1}^{K} \hat{\omega}_{k}^{\left( n \right)} g\left( \mathbf{x};\hat{m}_{k}^{\left( n \right)},\hat{\sigma}_{k}^{\left( n \right)} \right)L_{h}\left( \boldsymbol{\Phi,x} \right|\omega_{1},\cdots,\omega_{K}, m_{1},\cdots,m_{K},\sigma_{1},\cdots,\sigma_{K} )d\mathbf{x}=:Q_{\boldsymbol{\Phi}}\left( \boldsymbol{\theta} , {\hat{\boldsymbol{\theta}}}^{(n)} \right)$, (S1.7)

where $\boldsymbol{\theta}$ and ${\hat{\boldsymbol{\theta}}}^{(n)}$ are the vectors of hyperparameters, such that

$\left\{ \begin{aligned} {\boldsymbol{\theta}=(\omega}_{1},\cdots,\omega_{K}, m_{1},\cdots,m_{K},\sigma_{1},\cdots,\sigma_{K}) , \\ {\hat{\boldsymbol{\theta}}}^{(n)}=\left( \hat{\omega}_{1}^{\left( n \right)},\cdots,\hat{\omega}_{K}^{\left( n \right)}, \hat{m}_{1}^{\left( n \right)},\cdots,\hat{m}_{K}^{\left( n \right)},\hat{\sigma}_{1}^{\left( n \right)},\cdots,\hat{\sigma}_{K}^{\left( n \right)} \right) for n\in positive integers, \end{aligned} \right.$(S1.8)

and $\mathbf{x}$ represents a random variable.

- M-step: Determine the parameters, ${\hat{\boldsymbol{\theta}}}^{(n+1)},$ such that

${\hat{\boldsymbol{\theta}}}^{(n+1)}=\arg\max_{\boldsymbol{\theta}} Q_{\boldsymbol{\Phi}}\left( \boldsymbol{\theta} , {\hat{\boldsymbol{\theta}}}^{(n)} \right)$. (S1.9)

In order to find the set of hyperparameters, $\theta$, the following recursive relationship of elements in ${\hat{\boldsymbol{\theta}}}^{(n)}$ is obtained:

$${\hat{\omega}_{i}}^{\left( n+1 \right)}= \frac{1}{N}\sum_{j}^{N} z_{ij}^{\left( n \right)},$$

${\hat{m}_{i}}^{(n+1)}= \frac{\sum_{j}^{N} z_{ij}^{(n)}x_{j}}{\sum_{j}^{N} z_{ij}^{(n)}}$, and (S1.10)

$\left( {\hat{\sigma_{i}}}^{\left( n+1 \right)} \right)^{2}= \frac{\sum_{j}^{N} z_{ij}^{(n)} {{(x}_{j}- \hat{m}_{i}^{\left( m+1 \right)})}^{2}}{\sum_{j}^{N} z_{ij}^{(n)}}$,

where

$z_{ij}^{(n)}:=\frac{\hat{\omega}_{i}^{\left( n \right)} g(x_{j};\hat{m}_{i}^{(n)},\hat{\sigma}_{i}^{(n)})}{\sum_{k=1}^{K} \hat{\omega}_{k}^{\left( n \right)} g(x_{j};\hat{m}_{k}^{\left( n \right)}, \hat{\sigma}_{k}^{(n)})}$ . (S1.11)

- Continue to perform the E- step and the M-step until a positive, infinitesimal number, $\epsilon,$ exists, such that

$\left| {\hat{\boldsymbol{\theta}}}^{(n)}-{\hat{\boldsymbol{\theta}}}^{(m)} \right|<\epsilon$, (S1.12)

for $n>N,$ where N is an actual large number.

**Example calculation of K–L divergence from CHEMBL539392 as a query ligand.**

Using the above equation (2.6) in the main text for Kullback–Leibler divergence between normal distributions,

$D\left( G\left( x;m_{i}, \sigma_{i} \right)\left\| G\left( x;m_{j}, \sigma_{j} \right) \right. \right)=\ln\left( \frac{\sigma_{j}}{\sigma_{i}} \right)+\frac{\left( \sigma_{i} \right)^{2}+\left( m_{i}-m_{j} \right)^{2}}{2\left( \sigma_{j} \right)^{2}}-\frac{1}{2} ,$(3.14)

where

$\left\{ \begin{aligned} G\left( x;m_{i}, \sigma_{i} \right)=\Xi_{ML}(\phi_{1n}^{\left( 1 \right)}(x)|g, \omega, \mu, \sigma, 1) \\ G\left( x;m_{j}, \sigma_{j} \right)=\Xi_{EM}(\phi_{n}(x)|g, \omega, \mu, \sigma, 1) \end{aligned} \right.$ , (3.15)

When *n* = 1, i.e., the class was ESR, the parameters were as follows:

$m_{i}=0.24055, \sigma_{i}=0.07472, m_{j}=0.5483, \sigma_{j}=0.1458$*,*  (S2.1)

and the following K–L divergence was obtained:

$D\left( G\left( x;m_{i}, \sigma_{i} \right)\left\| G\left( x;m_{j}, \sigma_{j} \right) \right. \right)=2.1493$. (S2.2)

When *n* = 2, i.e., the class was VDR, the parameters were as follows:

$m_{i}=0.21976, \sigma_{i}=0.06466, m_{j}=0.5981, \sigma_{j}=0.1224$, (S2.3)

and the following K–L divergence was obtained:

$D\left( G\left( x;m_{i}, \sigma_{i} \right)\left\| G\left( x;m_{j}, \sigma_{j} \right) \right. \right)=4.6939.$ (S2.4)

When *n* = 3, i.e., the class was Cyclooxygenase-2, the parameters were as follows:

$m_{i}=0.24389, \sigma_{i}=0.04857, m_{j}=0.5941 \sigma_{j}=0.1758,$ (S2.5)

and the following K–L divergence was obtained:

$D\left( G\left( x;m_{i}, \sigma_{i} \right)\left\| G\left( x;m_{j}, \sigma_{j} \right) \right. \right)=2.0810.$ (S2.6)

When n = 4, i.e., the class was CTSD, the parameters were as follows:

$m_{i}=0.21187, \sigma_{i}=0.06631, m_{j}=0.4560, \sigma_{j}=0.1320$, (S2.7)

and the following K–L divergence was obtained:

$D\left( G\left( x;m_{i}, \sigma_{i} \right)\left\| G\left( x;m_{j}, \sigma_{j} \right) \right. \right)=1.6354.$(S2.8)

**S.Table 1.** Summary of datasets

| **Dataset** | **Original Conformers**  **(Compounds)** | **Sampled Conformers**  **(Compounds)** | **Resource** |
| --- | --- | --- | --- |
| Estrogen receptor alpha (ESR) | 278,809  (3460) | 13,957  (3,460) | ChEMBL23 |
| Vitamin D receptor (VDR) | 1,395,788  (11,715) | 13,957  (11,715) | ChEMBL23 |
| Cyclooxygenase-2 (COX2) | 281,176  (3,736) | 13,957  (3,736) | ChEMBL23 |
| Cathepsin D (CTSD) | 154,129  (856) | 13,957  (856) | ChEMBL23 |
| HIV-1 protease (HIV1) | 2,366,517  (603) | 13,957  (603) | ChEMBL25 |
| Heat shock protein 90 (HSP90) | 72,578  (459) | 13,957  (459) | ChEMBL25 |
| Transient receptor potential cation channel subfamily V4 (TRPV4) | 74,591  (468) | 13,957  (468) | ChEMBL25 |
| DNA topoisomerase I (TOP1) | 47,957  (282) | 13,957  (282) | ChEMBL25 |

**S.Table2.** The overlapped ligands between target class

|  | ESR | VDR | COX2 | CTSD | HIV1 | HSP90 | TRPV4 | TOP1 |
| --- | --- | --- | --- | --- | --- | --- | --- | --- |
| ESR | self | 281 | 634 | 2 | 5 | 0 | 0 | 7 |
| VDR | 281 | self | 130 | 1 | 7 | 4 | 0 | 4 |
| COX2 | 634 | 130 | self | 2 | 7 | 2 | 1 | 8 |
| CTSD | 2 | 1 | 2 | self | 4 | 0 | 0 | 0 |
| HIV1 | 5 | 7 | 7 | 4 | self | 0 | 0 | 0 |
| HSP90 | 0 | 4 | 2 | 0 | 0 | self | 0 | 1 |
| TRPV4 | 0 | 0 | 1 | 0 | 0 | 0 | self | 0 |
| TOP1 | 7 | 4 | 8 | 0 | 0 | 1 | 0 | self |

**Table3.** Structure information of randomly chosen queries for K-L divergence calculation from 4 targets (ESR, VDR, COX2, and CTSD).

| ID | CHEMBL539392 | CHEMBL193280 | CHEMBL443605 |
| --- | --- | --- | --- |
| ESR  Ligand | 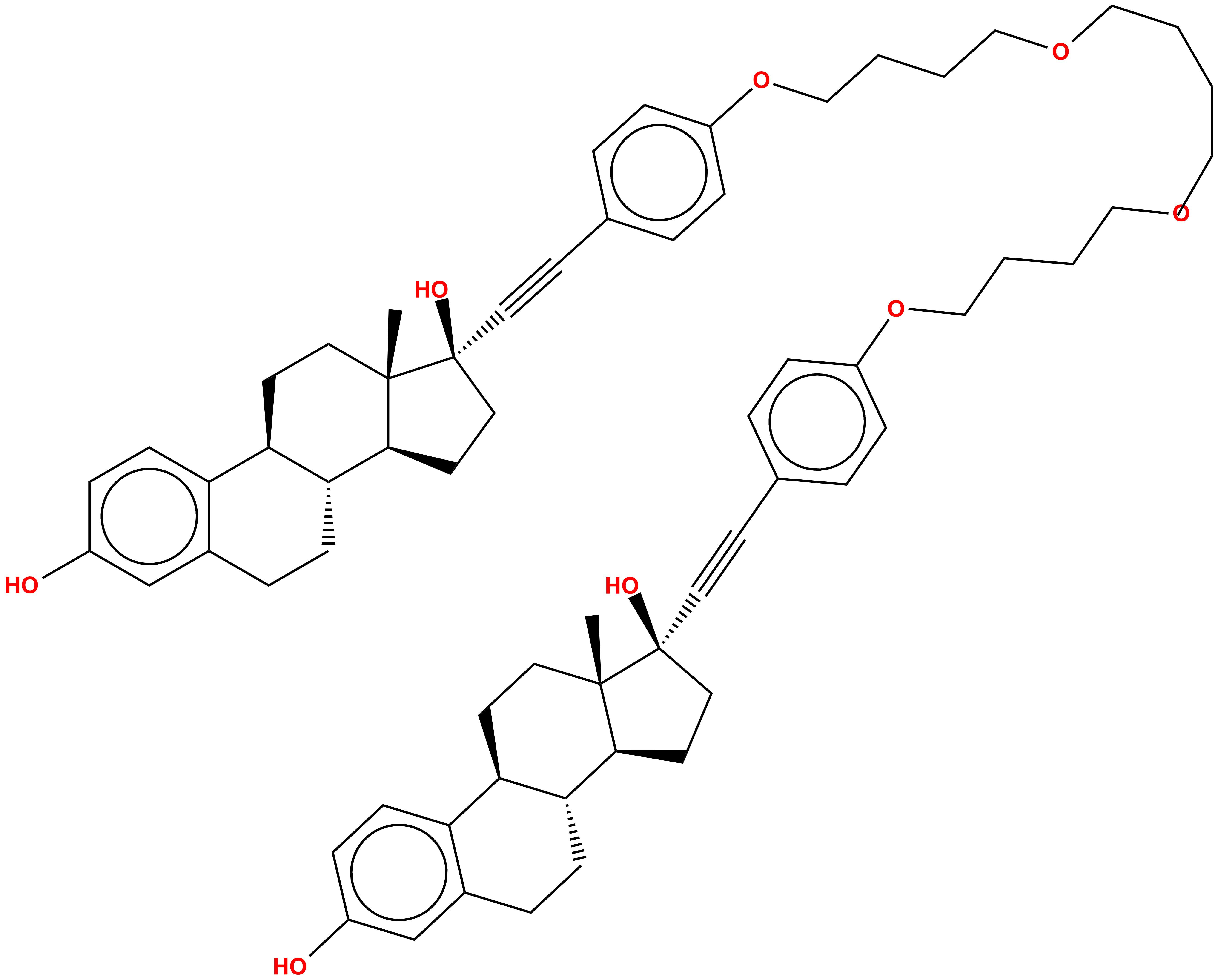 | 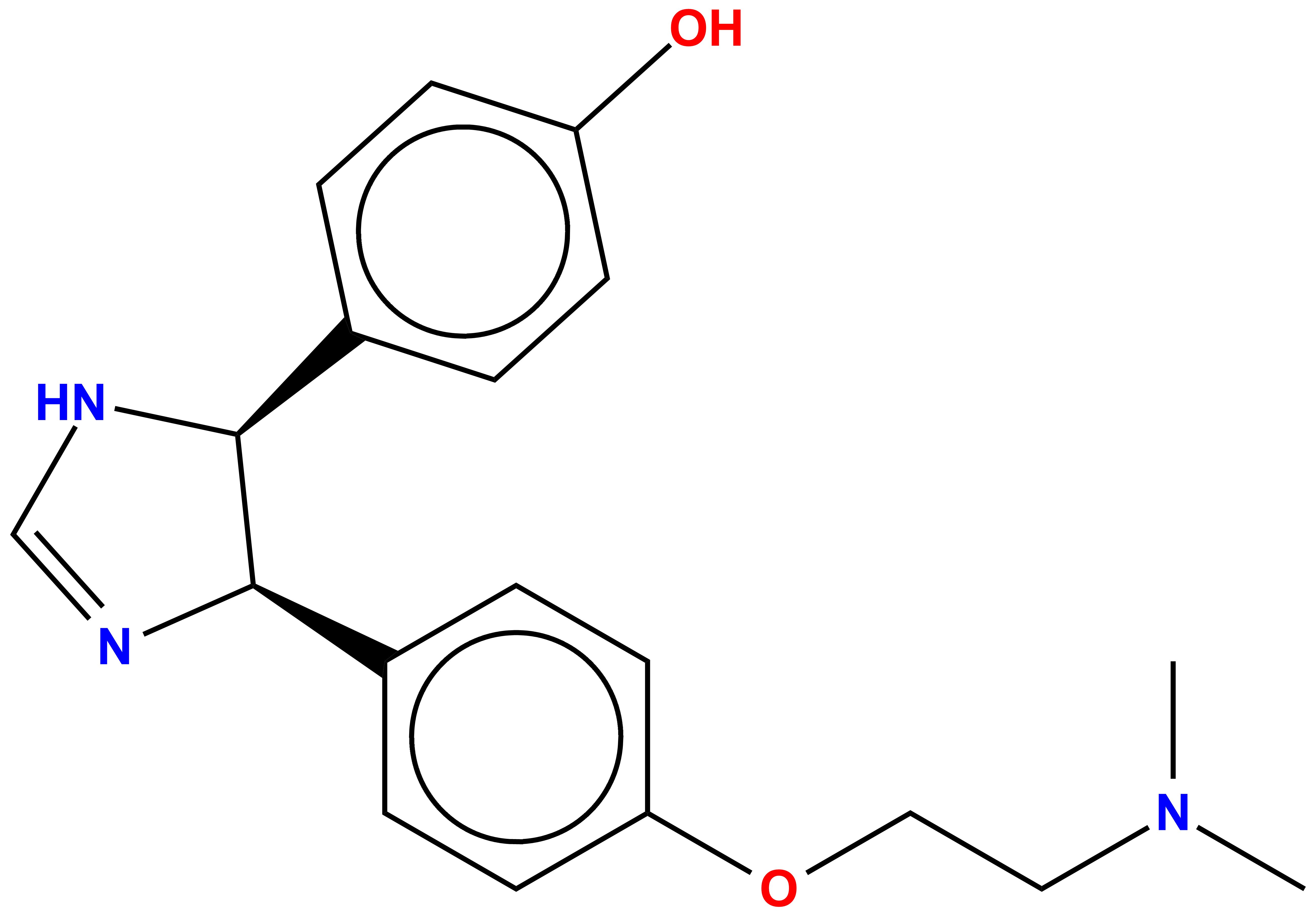 | 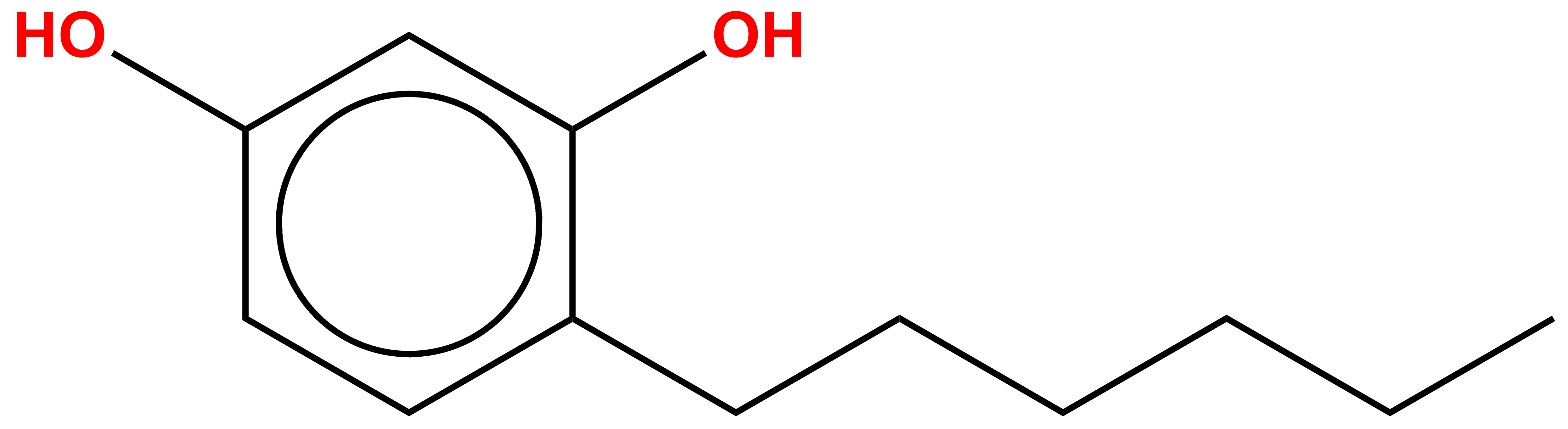 |
| ID | CHEMBL7162 | CHEMBL1322390 | CHEMBL1452735 |
| VDR  Ligand | 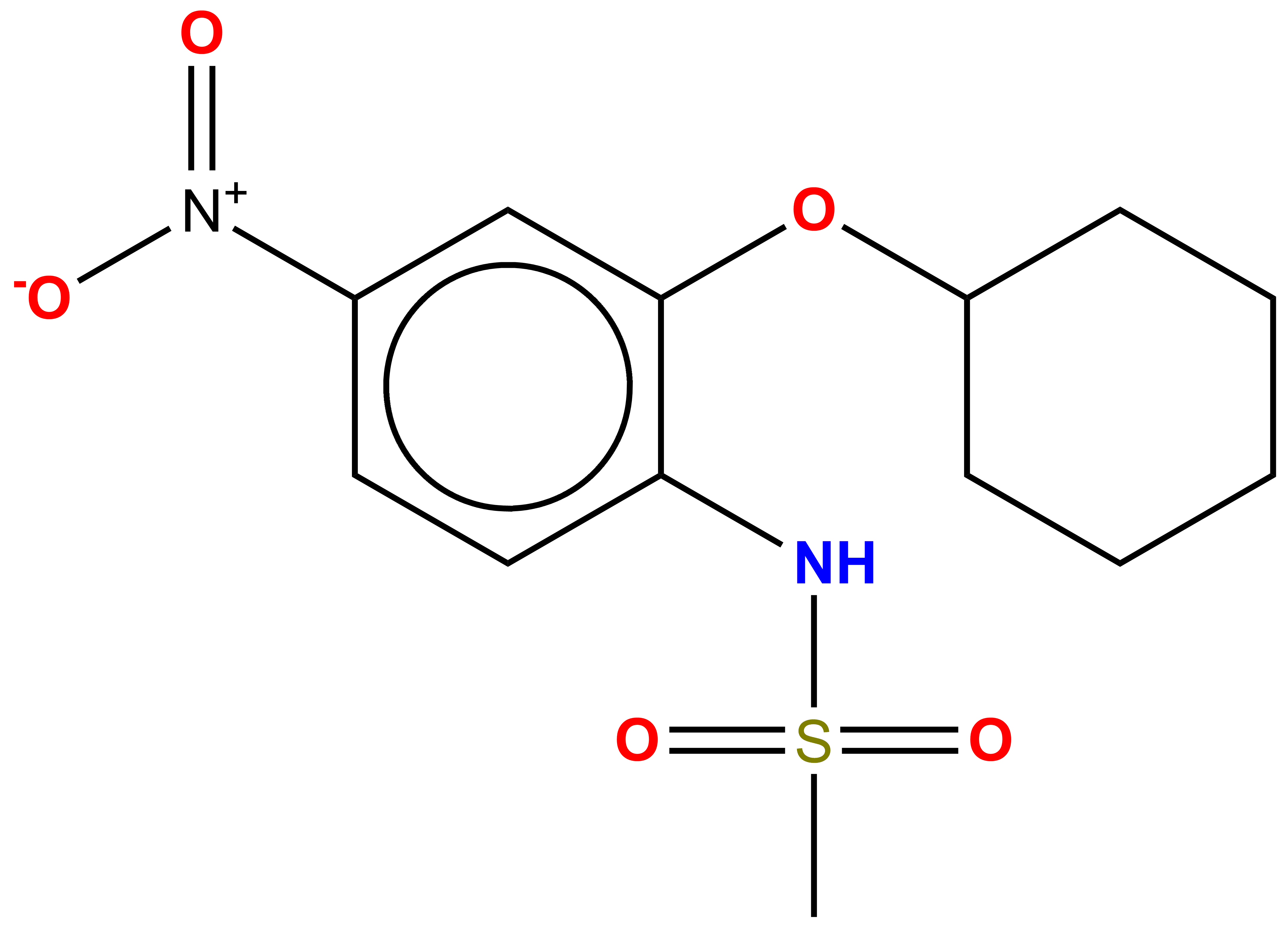 | 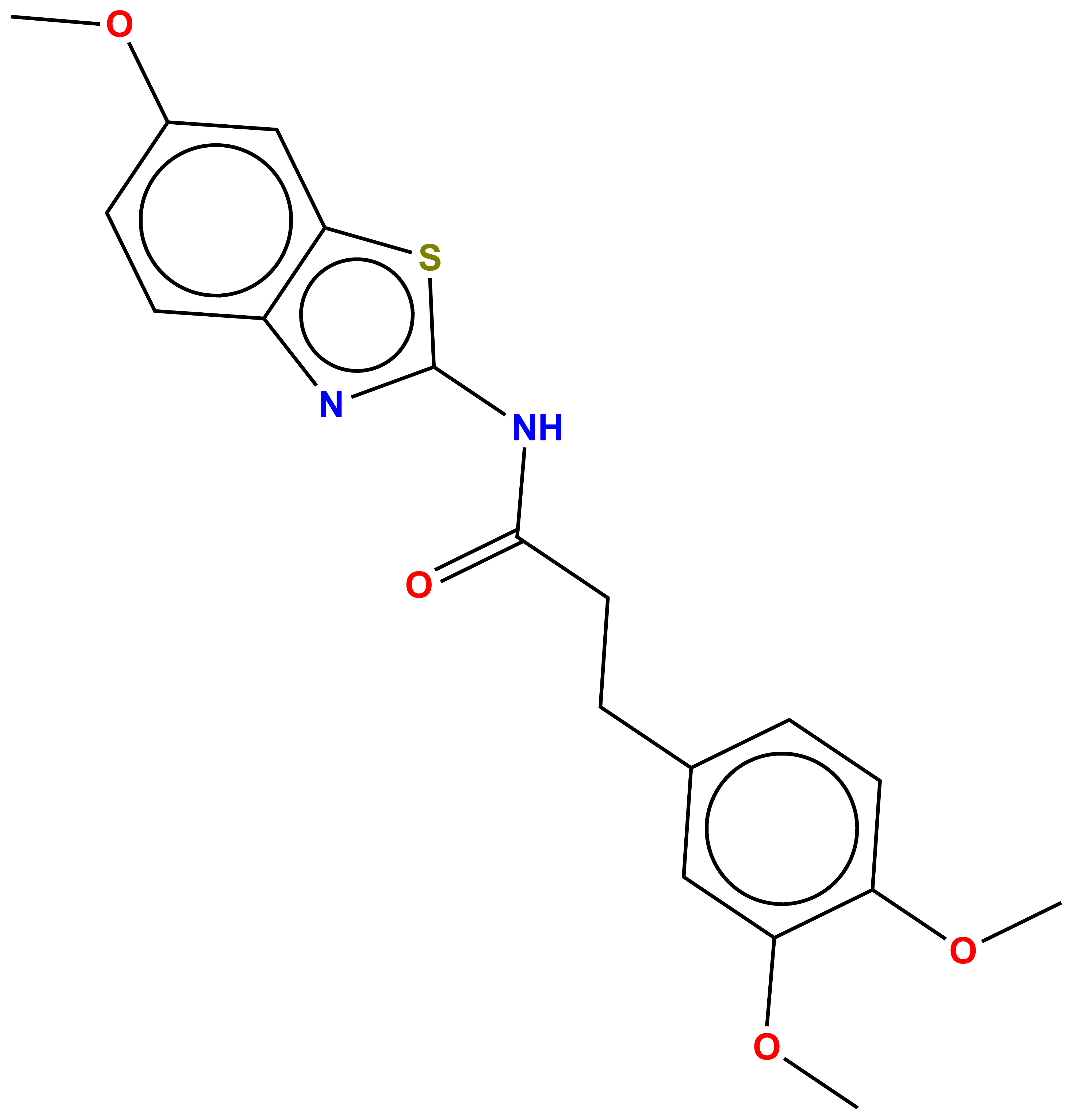 | 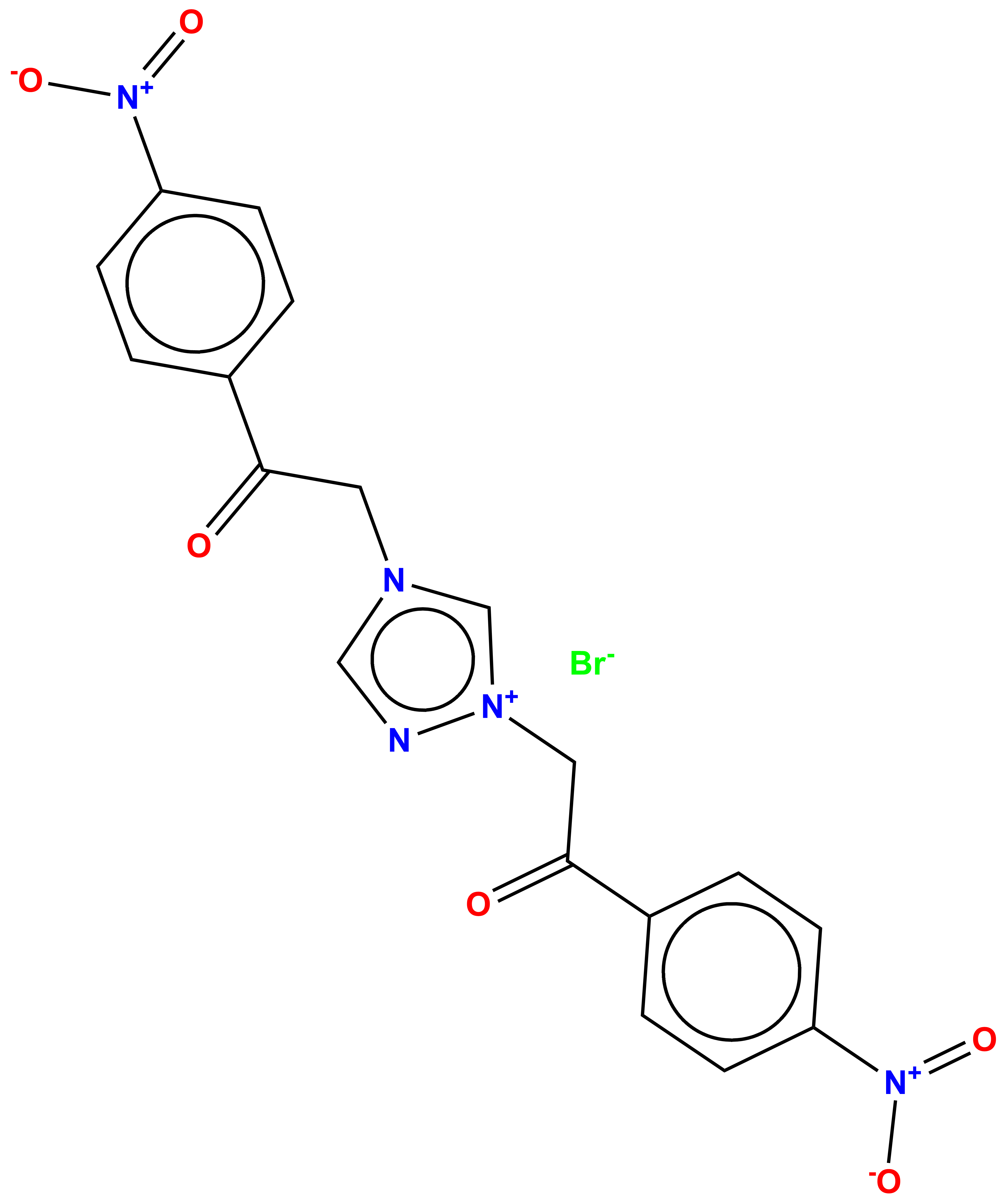 |
| ID | CHEMBL1163237 | CHEMBL127560 | CHEMBL271614 |
| COX2  Ligand | 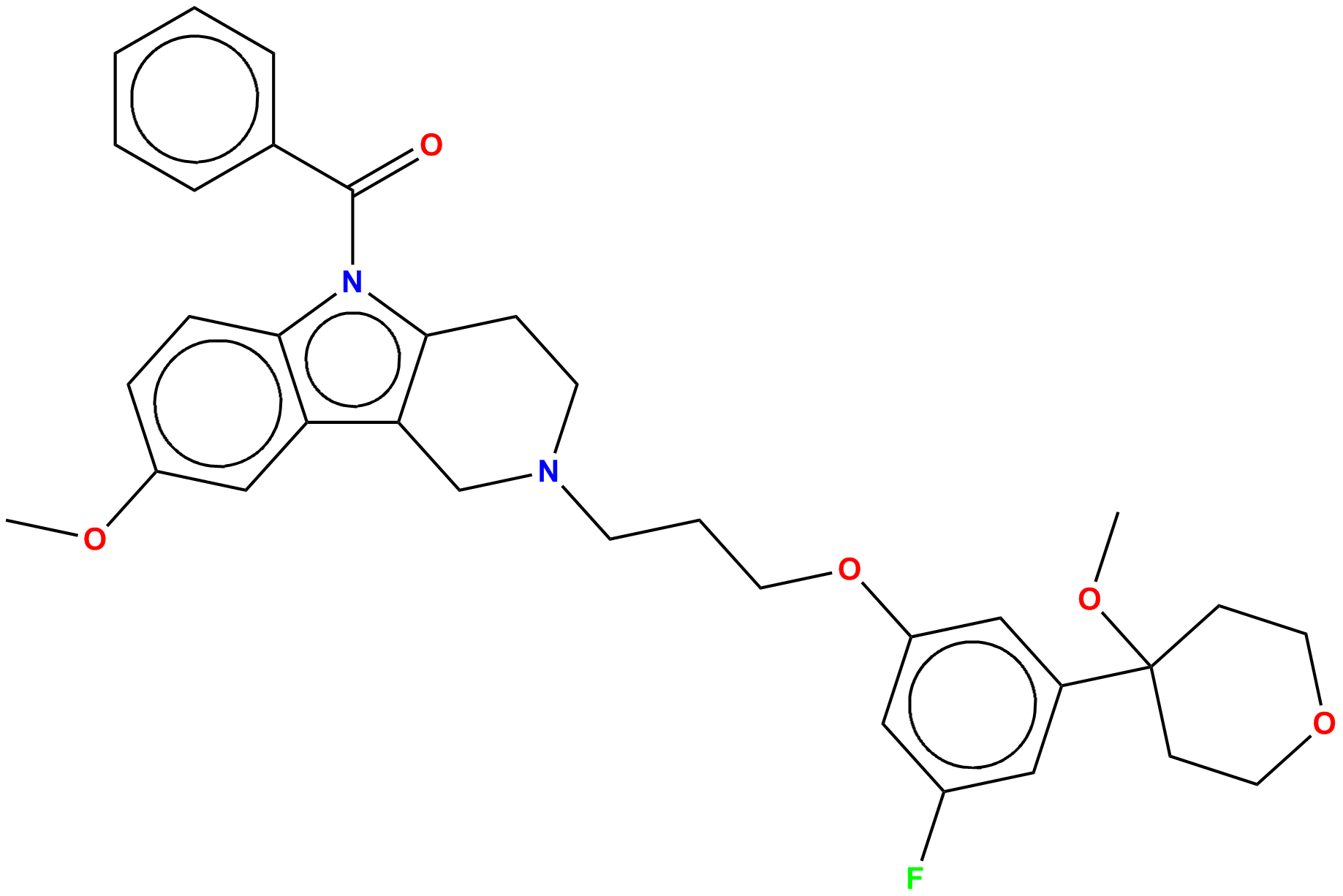 | 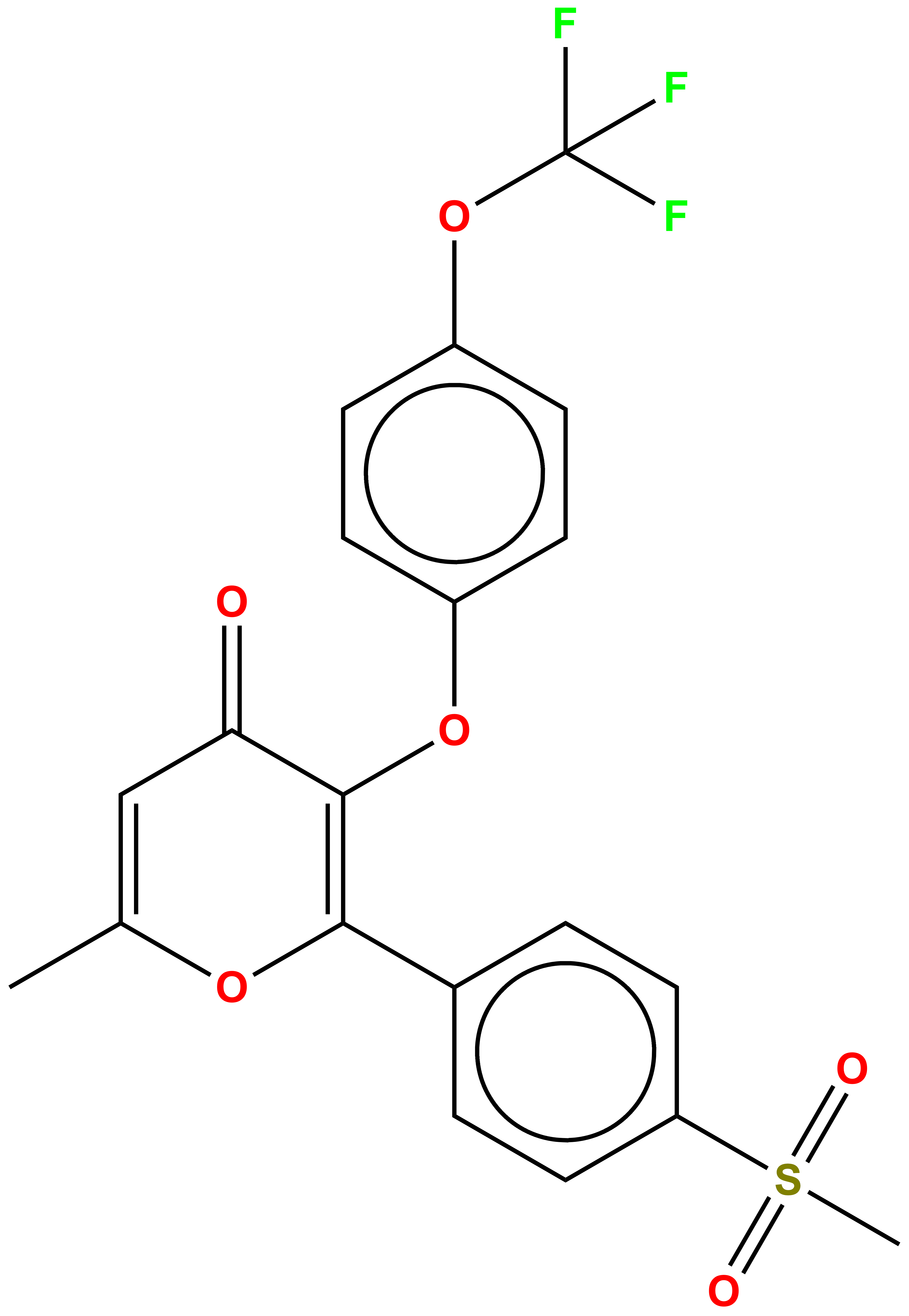 | 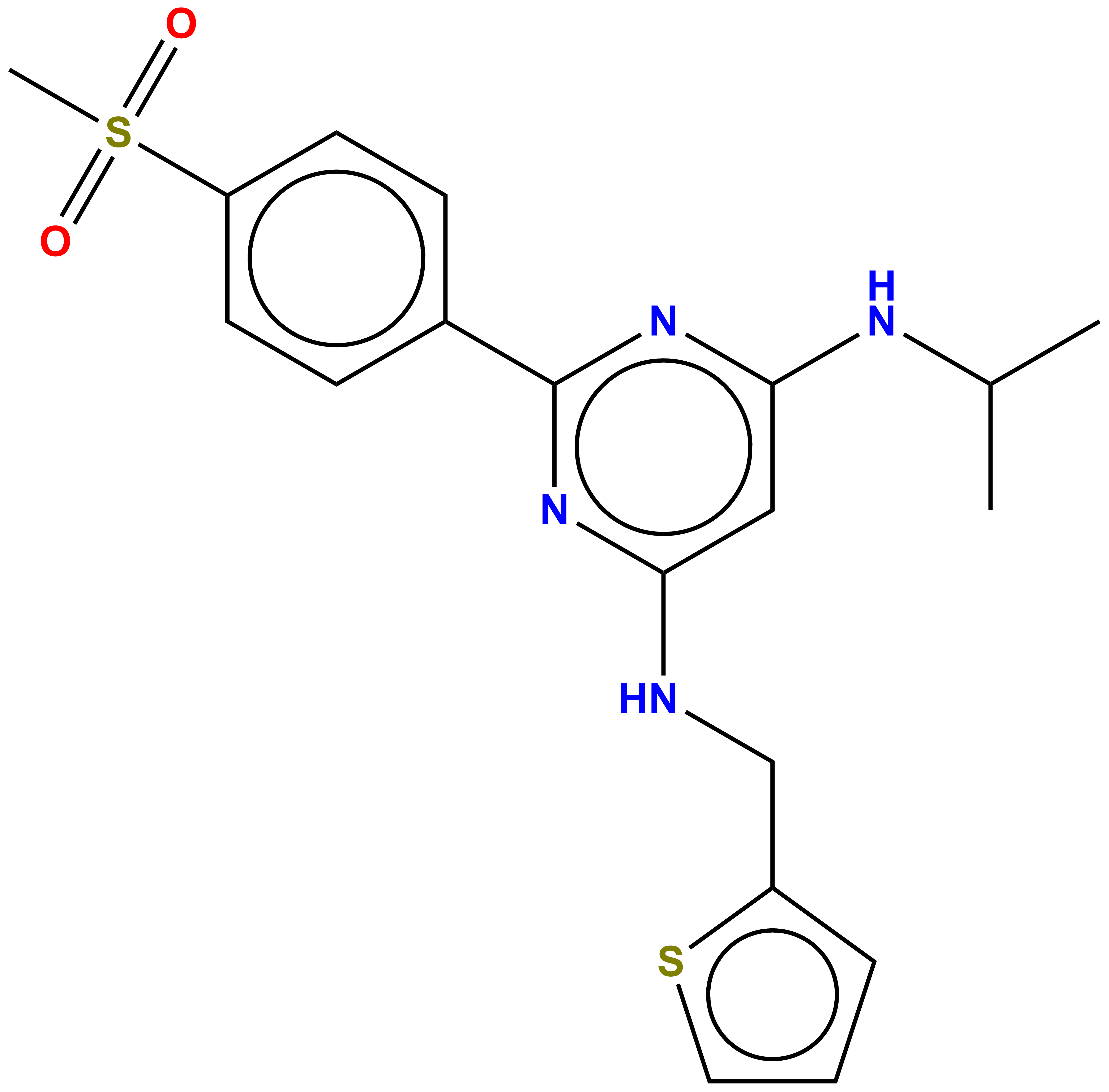 |
| ID | CHEMBL263810 | CHEMBL252655 | CHEMBL436438 |
| CTSD  Ligand | 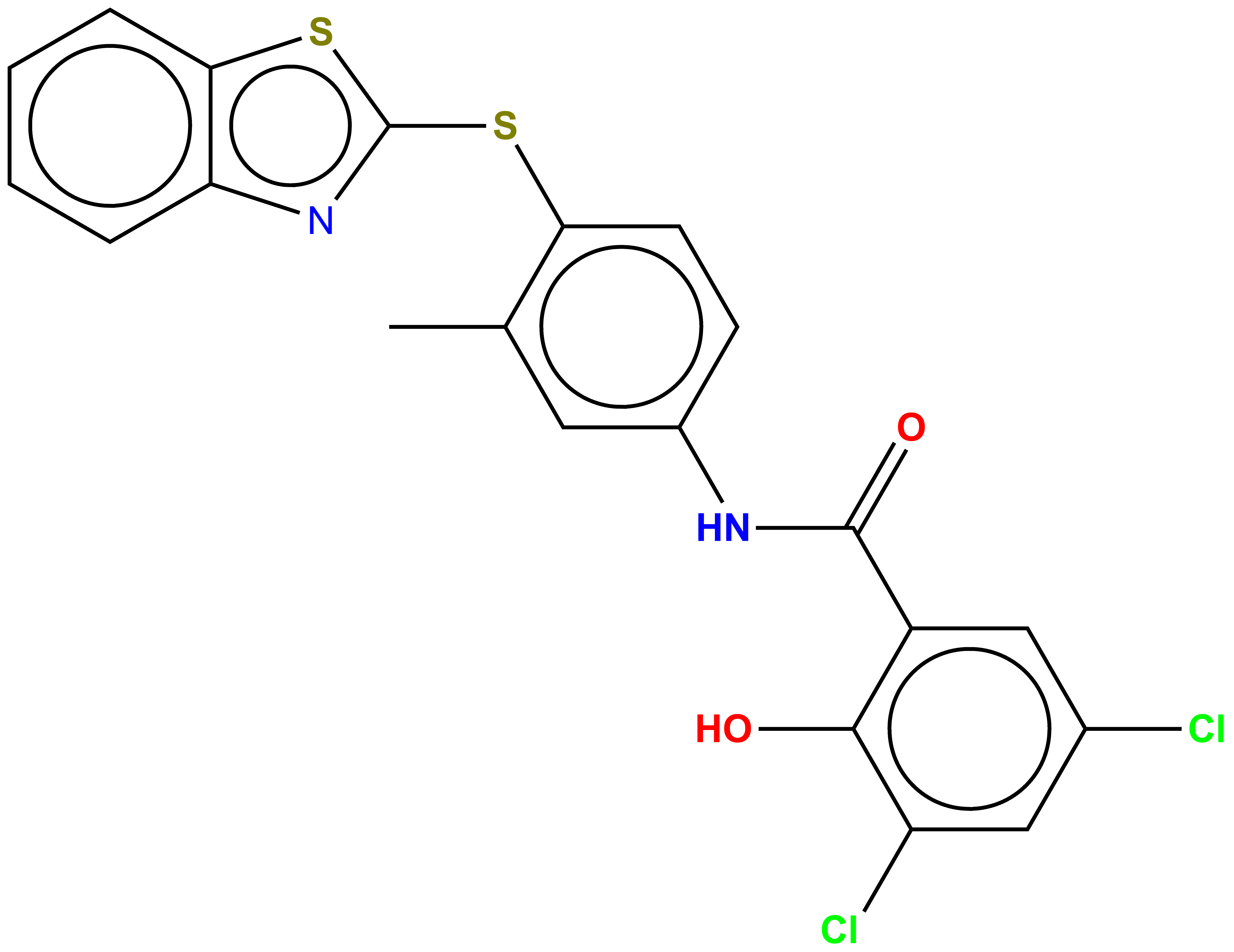 | 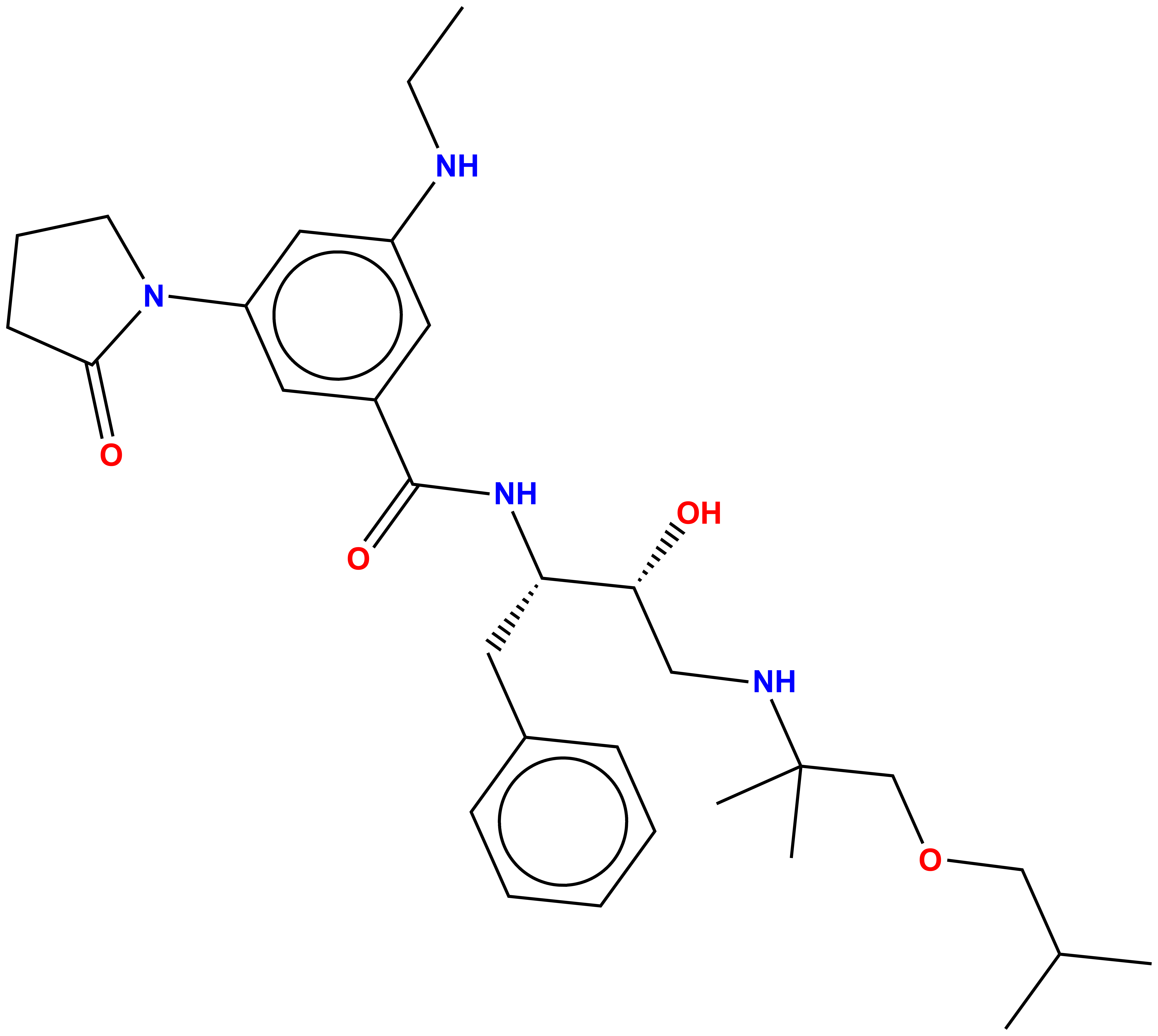 | 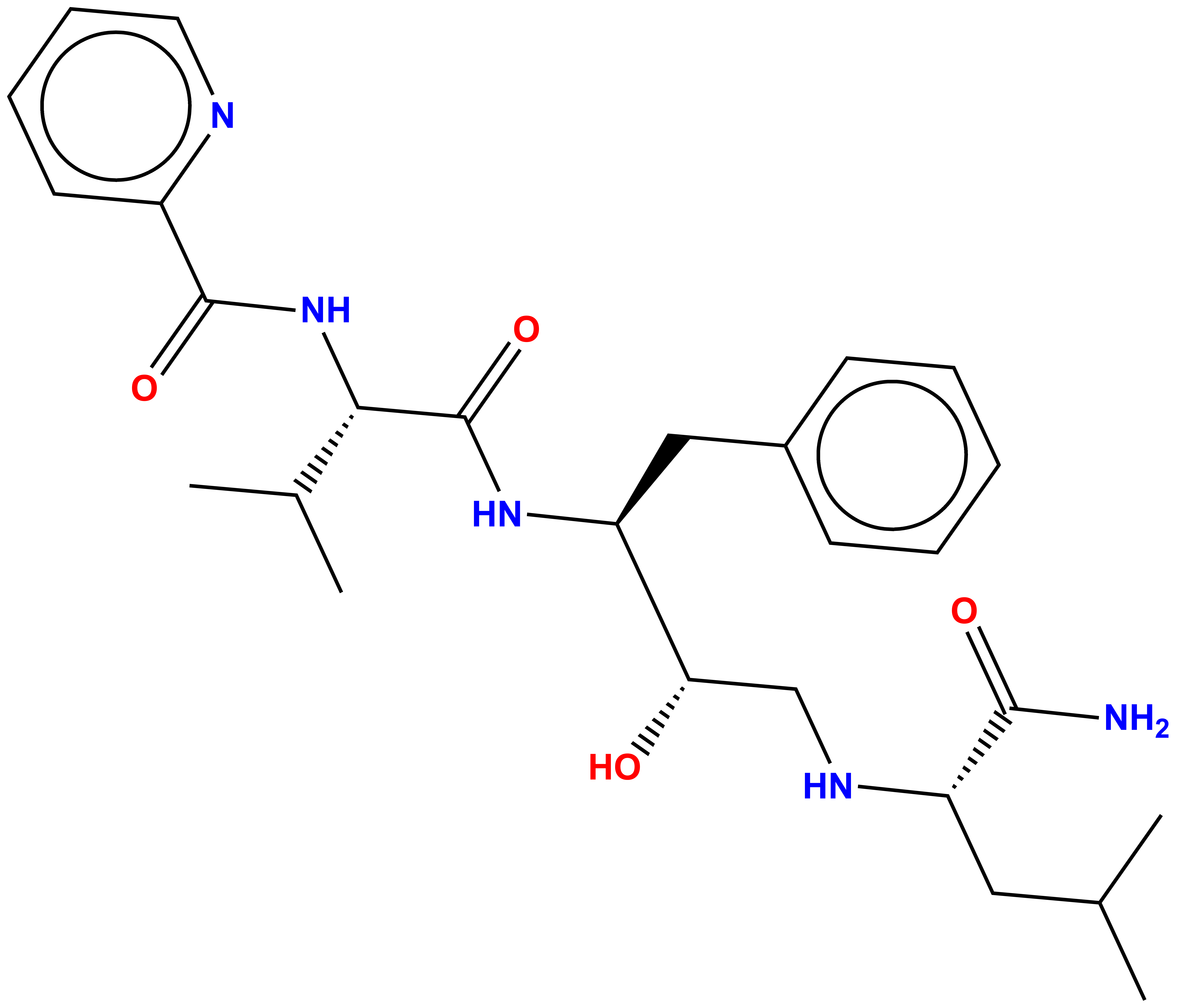 |

**S.Figure1.** *Q*-distribution of HIV1 based on Gaussian mixture model


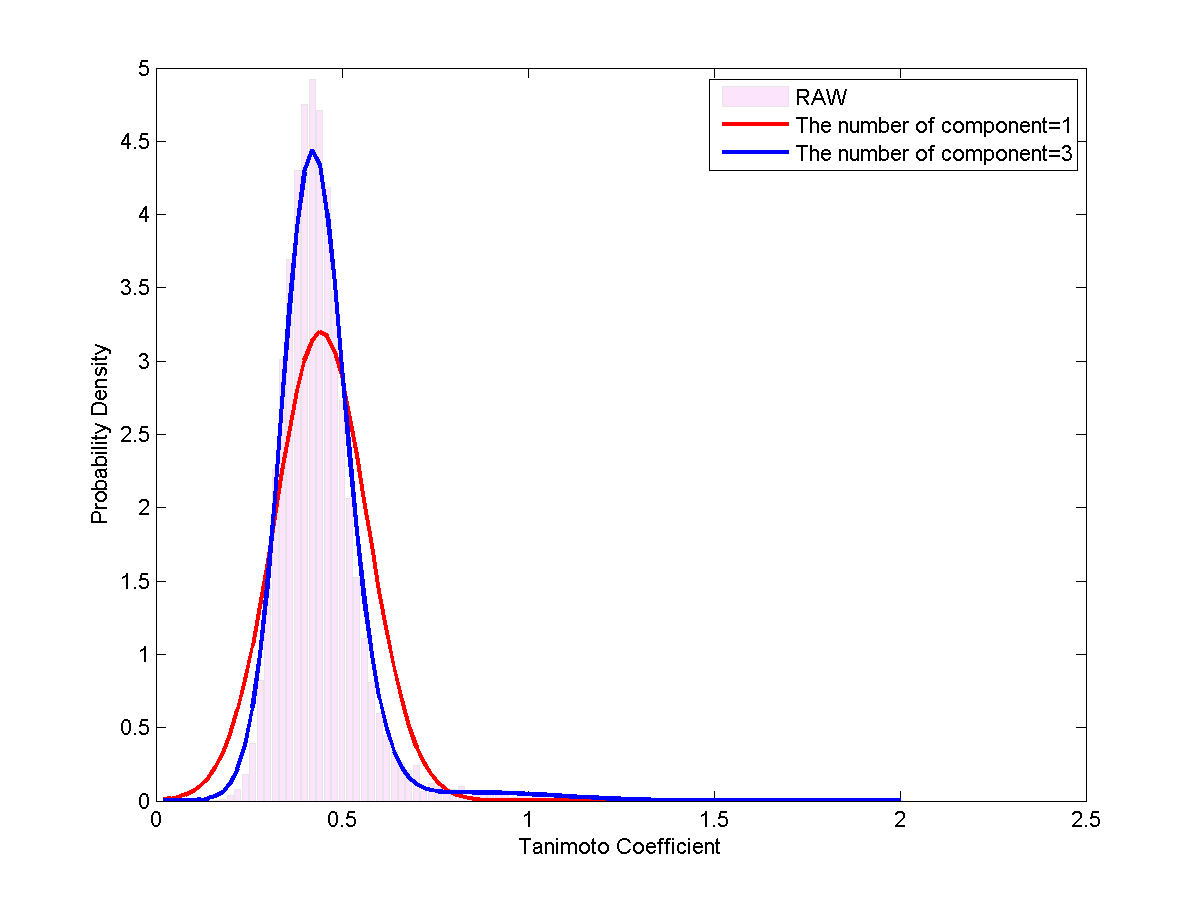


**S.Figure2.** *Q*-distribution of HSP90 based on Gaussian mixture model


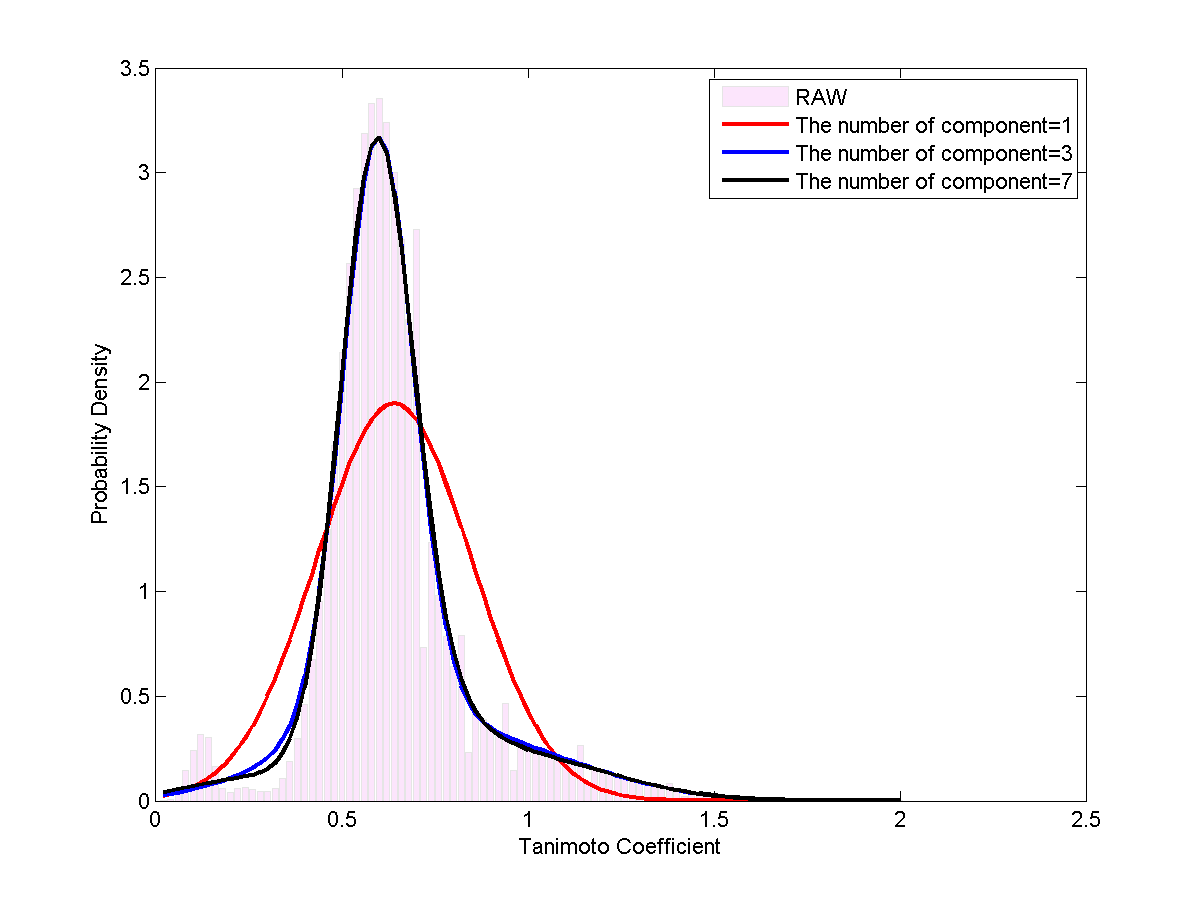


**S.Figure3.** *Q*-distribution of TRPV4 based on Gaussian mixture model


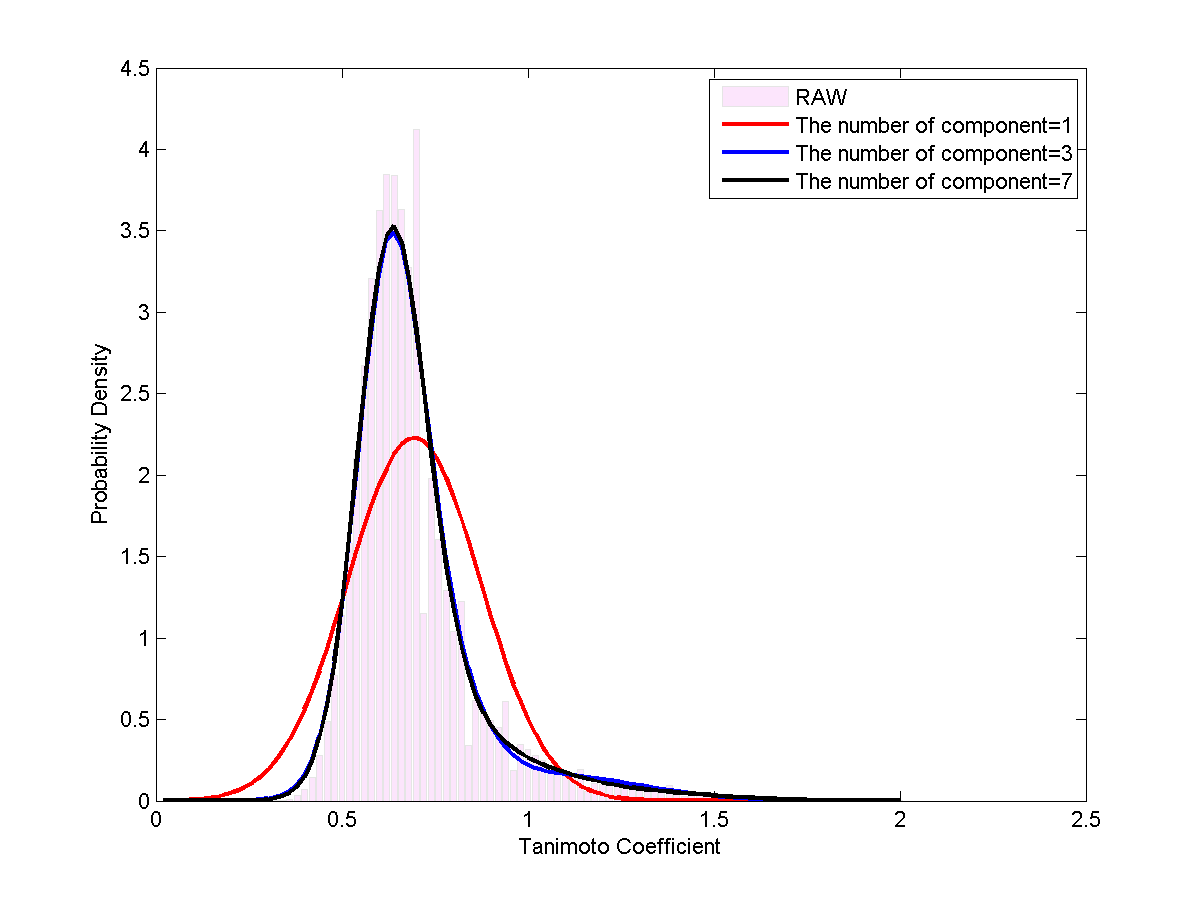


**S.Figure4.** *Q*-distribution of TOP1 based on Gaussian mixture model


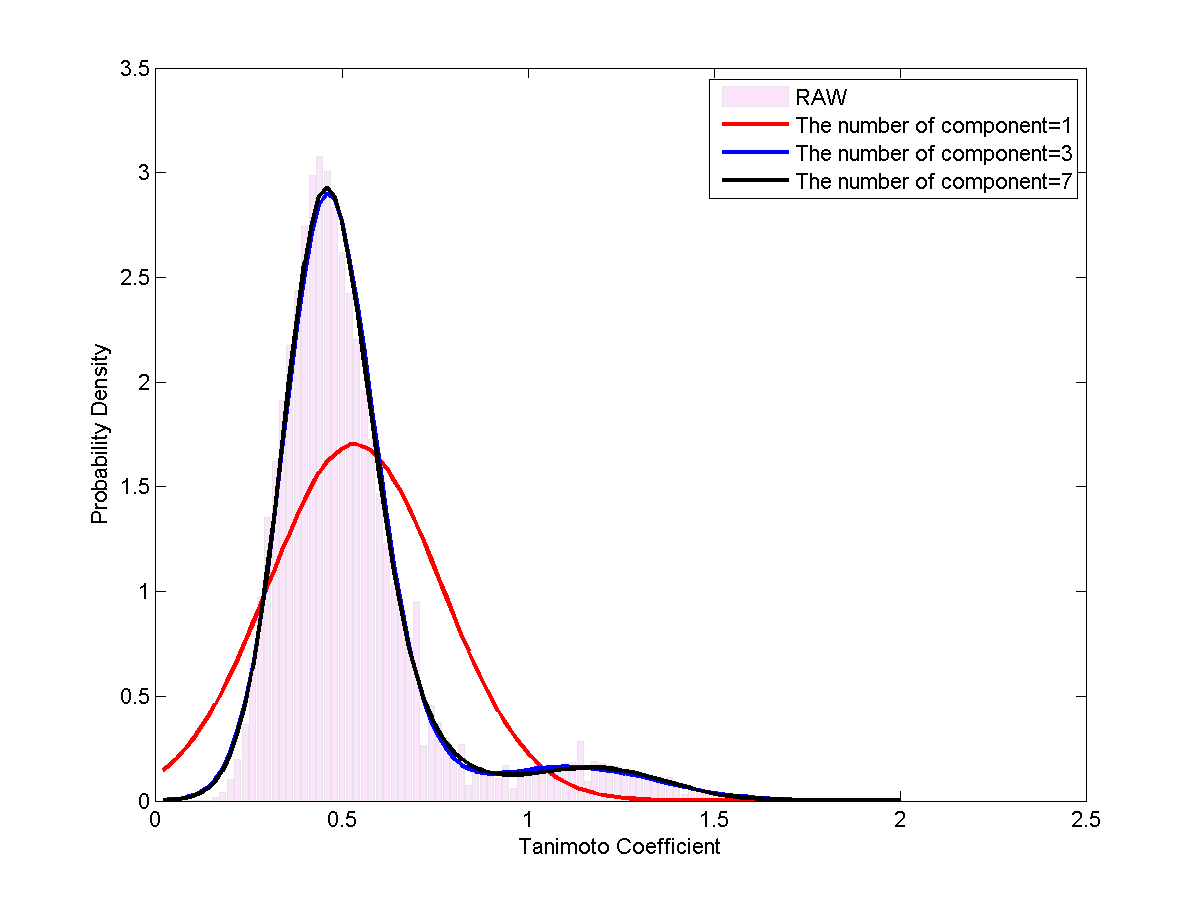


**S. Figure 5.** Frequency distributions of $\Xi_{ML}\left( \Phi_{1n}^{(l)}\left( x_{k} \right) | g,\omega,\mu, \sigma, 1 \right)$ estimates ($\mu_{1}$and$\sigma_{1}$). Query (*l*) $\in$ ESR (class = 1). (a) ESR-ESR, (b) ESR-VDR, (c) ESR-COX2, and (d) ESR-CTSD.* *The color bars (right side of the distribution) indicate frequency (eg. Yellow in 3(d) means that for over 3000 queries, the mean of the ML estimates varied from 0.45 to 0.5 and their standard deviation varied from 0.09 to 0.11.).


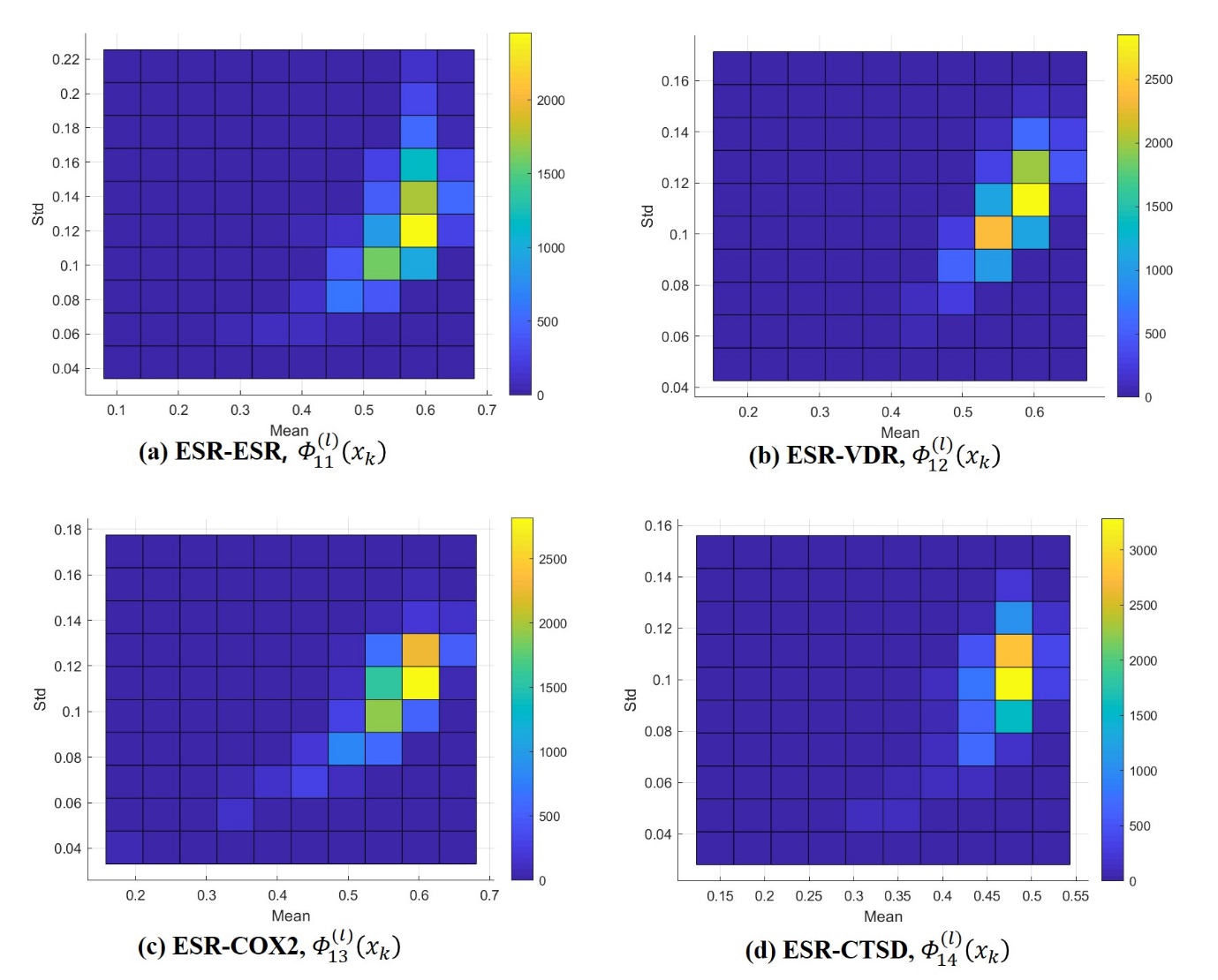


**S. Figure 6.** Frequency distributions of $\Xi_{ML}\left( \Phi_{2n}^{(l)}\left( x_{k} \right) | g,\omega,\mu, \sigma, 1 \right)$estimates ($\mu_{1}$and$\sigma_{1}$). Query (*l*) $\in$ VDR (class = 2). (a) VDR-ESR, (b) VDR-VDR, (c) VDR-COX2, and (d) VDR-CTSD.* *The color bars (right side of the distribution) indicate frequency (eg. Yellow in 4(d) means that for over 2500 queries, the mean of the ML estimates varied from 0.4 to 0.45 and their standard deviation varied from 0.052 to 0.062.).


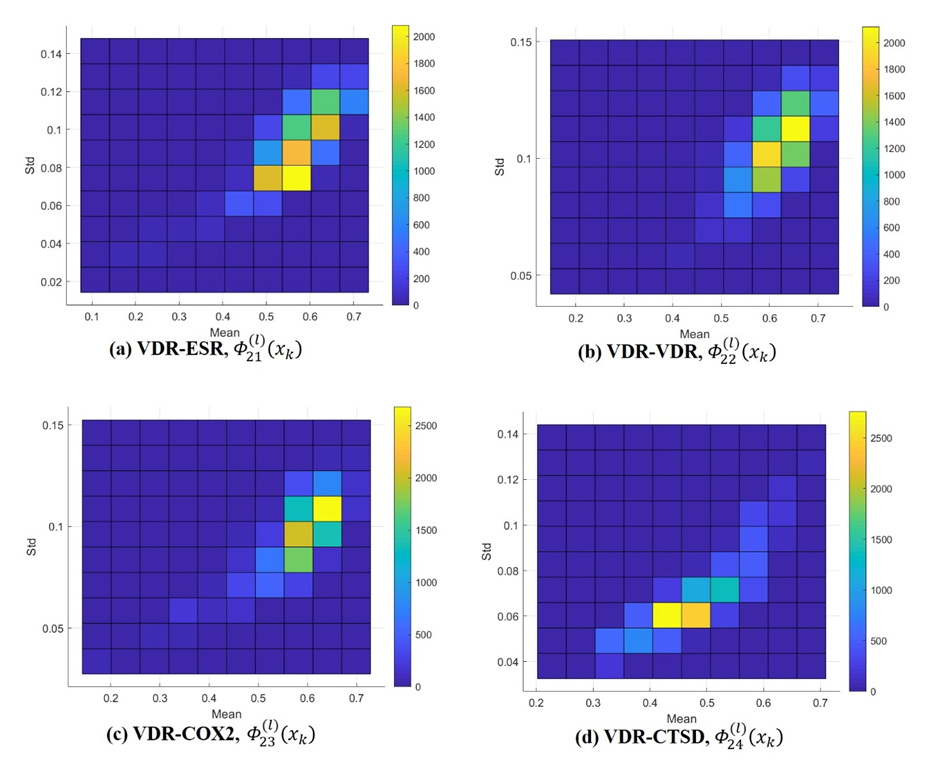


**S. Figure 7.** Frequency distributions of $\Xi_{ML}\left( \Phi_{3n}^{(l)}\left( x_{k} \right) | g,\omega,\mu, \sigma, 1 \right)$ estimates ($\mu_{1}$and$\sigma_{1}$). Query (*l*) $\in$ COX2 (class = 3). (a) COX2-ESR, (b) COX2-VDR, (c) COX2-COX2, and (d) COX2-CTSD.* *The color bars (right side of the distribution) indicate frequency (eg. Yellow in 5(a) means that for over 3500 queries, the mean of the ML estimates varied from 0.5 to 0.6 and their standard deviation varied from 0.1 to 0.12 in the standard.).


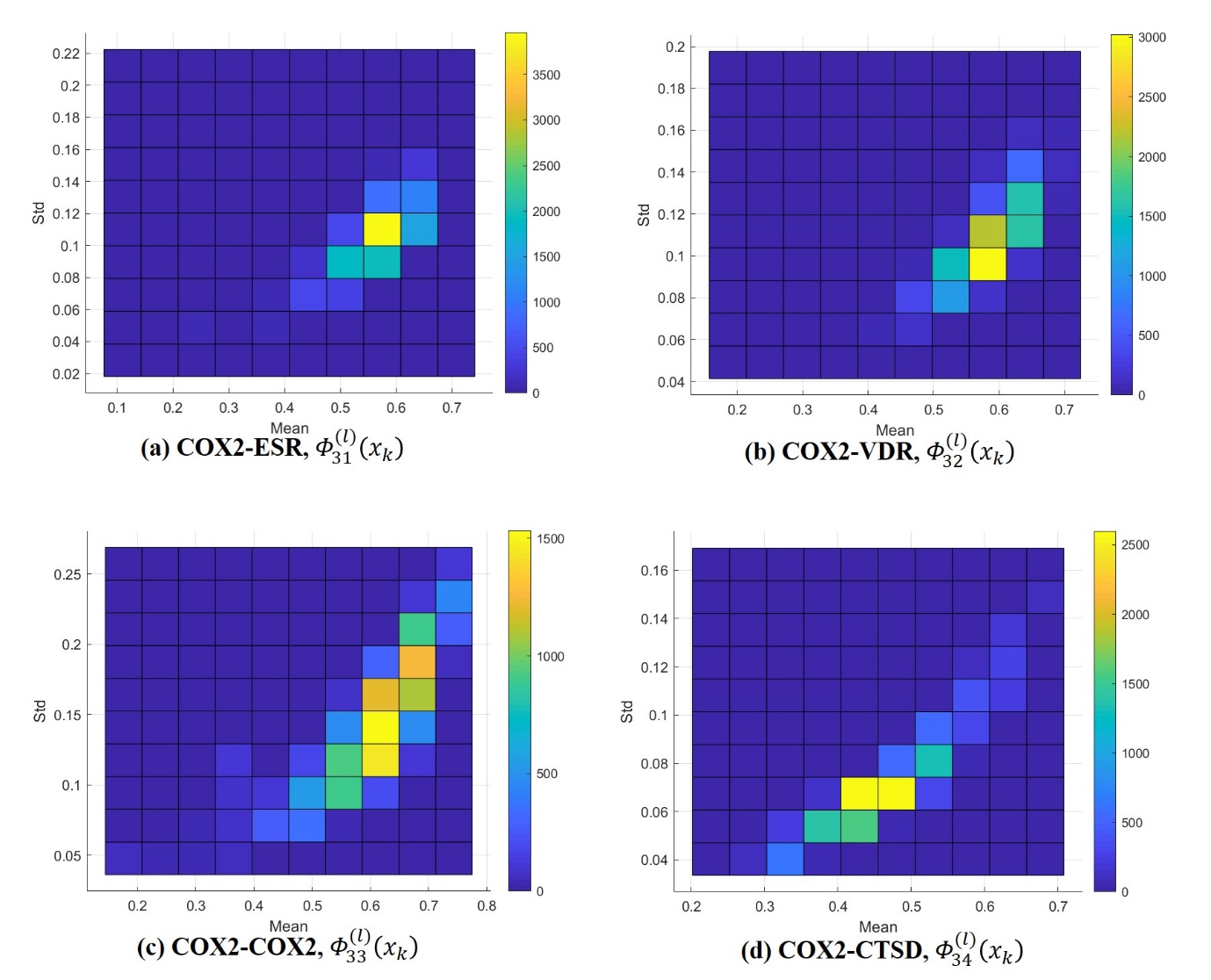


Python script (.py) for 3D similarity matrix

import sys

from openeye import oechem

from openeye import oeshape

from threading import Thread

def BConf(Aconf, prep, molB, csvresult):

csvresult.write("%s_%d" % (Aconf.GetTitle(), Aconf.GetIdx()))

refmol = oechem.OEGraphMol(Aconf)

options = oeshape.OEOverlayOptions()

options.SetOverlapFunc(oeshape.OEOverlapFunc())

overlay = oeshape.OEOverlay(options)

overlay.SetupRef(refmol)

bfs = oechem.oemolithread(molB)

fitmol = oechem.OEMol()

while oechem.OEReadMolecule(bfs, fitmol):

prep.Prep(fitmol)

scoreiter = oeshape.OEBestOverlayScoreIter()

oeshape.OESortOverlayScores(scoreiter, overlay.Overlay(fitmol), oeshape.OEHighestTanimotoCombo())

for score in scoreiter:

csvresult.write(",%.2f" % score.GetTanimotoCombo())

csvresult.write("\n")

def genHeader(filename, csvresult):

csvresult.write("name")

ifs = oechem.oemolithread(sys.argv[2])

mol = oechem.OEMol()

while oechem.OEReadMolecule(ifs, mol):

for conf in mol.GetConfs():

csvresult.write(",%s_%d" % (conf.GetTitle(), conf.GetIdx()))

csvresult.write("\n")

def main(argv=[__name__]):

if len(argv) != 4:

oechem.OEThrow.Usage("%s <Afile(Query_ref)> <Bfile_Fitmol> <csvresult>" % argv[0])

csvresult = open(argv[3], "w")

genHeader(sys.argv[2], csvresult)

prepA = oeshape.OEOverlapPrep()

afsA = oechem.oemolithread(sys.argv[1])

MA = afsA.GetOEMols()

for molA in MA:

prepA.Prep(molA)

confA= molA.GetConfs()

print(molA.GetTitle())

for conf in confA:

BConf(conf, prepA, sys.argv[2], csvresult)

if __name__ == "__main__":

import sys

sys.exit(main(sys.argv))
