# Supplementary material for: Comparing a Query Compound with Drug Target Classes Using 3D-Chemical Similarity
Source: Int J Mol Sci. 2020 Jun 12;21(12):4208. doi: 10.3390/ijms21124208 (PMC7352980; doi:10.3390/ijms21124208)
Supplement: Supplementary file 1 [file ijms-21-04208-s001.zip › 3.List-of-acronyms.docx]

Abbreviation and terminology list

#1. **‘Chemocentric assumption’** means two similar molecules are likely to have similar properties so two similar molecules can share biological targets or can show similar pharmacological profile.

#2. **‘Molecular framework’** means topological composition of a compound consisting of atoms and bonds, which generate rings, ring systems, and side-chains. For example, Bemis-Murcho framework is also a famous molecular framework. In drug discovery, ‘drug scaffold’ indicates molecular framework having drug-like properties. In particular, organic synthetic principles and core structure (generally, ring system) have been used to classify drug scaffolds. **‘Unprecedented molecular framework’** means new molecular framework different from existing molecular frameworks. Typically, a novel scaffold means distinct scaffold from known drug scaffolds within a target. However, ‘novel’ cannot mean ‘first existing’. ‘Unprecedented’ means ‘not existing before the first’.

#3. **‘maximum likelihood (ML) estimation’** is a method of estimating the parameters of a probability density distribution by maximizing a likelihood function, so that under the assumed statistical model the observed data is most probable. In this study, since the statistical model is normal distribution, the parameters are mean (μ) and standard deviation (σ). If sigma=sigma_1 and mu= mu_1, likelihood function is maximized and g(x: mu_1, sigma_1) is the most similar distribution with the distribution from observed data.

#4. **‘Gaussian mixture model (GMM)’** is a method that analyzes compositional data using mixed Gaussian distributions (one or multiple) as a probability density distribution. In general, GMM is used for clustering of data. At that time, one cluster means one Gaussian distribution.

#5. **‘expectation–maximization (EM) algorithm’** is an iterative method to find maximum likelihood or maximum a posteriori estimates of parameters in statistical models, where the model depends on unobserved latent variables.

#6. **‘discriminativeness’ in Kullback–Leibler (K–L) divergence:**

When two objects are compared, the goal of the comparison is finding different or common feature between them. The goal of Kullback–Leibler (K–L) divergence is measuring the difference but not the commonality. ‘discriminativeness’ is the condition comparing the difference. The words, ‘discriminate, discrimination’ also exist in the original article having Kullback and Leibler’s definition.

#7. **‘target class’:**

In drug discovery, ‘target class’ means higher hierarchy of targets such as nuclear receptor, GPCR, kinase, protease, ion channel. In this study, ‘target class’ is a group of chemical ligands annotated with one target. The terminology, ‘target class’ was used to escape the confusion between a target protein and ligands of a targets.

#8. **‘chemistry-oriented synthesis’** is a chemocentric approach for drug discovery.

Current drug discovery commonly uses ‘targets’ as their queries so that ‘structural uniqueness or diversity of drugs’ cannot be considered with either the highest priority or the uncoupling with SAR. However, a part of ‘not existing compounds’ can be unfound treasure island. For the purpose, ‘ChOS (Chemistry oriented synthesis)’ without any chosen target was devised to find unprecedented drug scaffolds with structural uniqueness. ChOS consists of four stepwise components; (1) the development of efficient synthetic methodology of an unprecedented druglike scaffold, (2) small diverse chemical library of the scaffold from the reaction methodology, (3) in silico target screening of the library, and (4) in vitro activity check against selected targets through in silico.

#9. **‘compound-target association’:**

A compound can directly bind to the target (docking) and other compounds can regulate the target indirectly. In addition, some compound regulates a function of the target and another can regulate expression level of target protein. The mentioned everything is included in “compound-target association”. In this study, dataset was extracted based on single target information but regardless of assay type (eg. cell-level, biochemical level, functional study).

#10. **‘Jaccard-Tanimoto similarity of query-to-ligand pairs’** is similarity coefficients between a query and ligands of a target class.

If a query exists in the *l*th column vector of the similarity matrix $\mathbf{T}_{\mathbf{M}}\left[ \mathbf{C}_{\mathbf{l}}\left( m \right), \mathbf{C}_{\mathbf{l}}\left( n \right) \right]_{ij}$:

$$\boldsymbol{\tau}_{\boldsymbol{m}}\left( m, n, l \right):=\mathbf{T}_{\mathbf{M}}\left[ \mathbf{C}_{\mathbf{l}}\left( m \right), \mathbf{C}_{\mathbf{l}}\left( n \right) \right]\mathbf{E}_{l}$$

$$m:the target class of query$$

$$n:the target class of ligands$$

When the matrix is divided according to each query, the number of matrixes for query-to-ligand pairs is No. (target)* No. (query) and the dimension of a matrix for query-to-ligand pairs: 1* 14,000 (if the query has one conformer).
